# A global bibliometric and visualized analysis of gait analysis and artificial intelligence research from 1992 to 2022

**DOI:** 10.3389/frobt.2023.1265543

**Published:** 2023-11-17

**Authors:** Tong Bao, Jiasi Gao, Jinyi Wang, Yang Chen, Feng Xu, Guanzhong Qiao, Fei Li

**Affiliations:** ^1^ School of Medicine, Tsinghua University, Beijing, China; ^2^ Institute for Precision Medicine, Tsinghua University, Beijing, China; ^3^ Orthopedics Department of the First Affiliated Hospital of Tsinghua University, Beijing, China; ^4^ Institute for AI Industry Research, Tsinghua University, Beijing, China

**Keywords:** gait analysis, artificial intelligence (AI), wearable device, sensor, bibliometric analysis

## Abstract

Gait is an important basic function of human beings and an integral part of life. Many mental and physical abnormalities can cause noticeable differences in a person’s gait. Abnormal gait can lead to serious consequences such as falls, limited mobility and reduced life satisfaction. Gait analysis, which includes joint kinematics, kinetics, and dynamic Electromyography (EMG) data, is now recognized as a clinically useful tool that can provide both quantifiable and qualitative information on performance to aid in treatment planning and evaluate its outcome. With the assistance of new artificial intelligence (AI) technology, the traditional medical environment has undergone great changes. AI has the potential to reshape medicine, making gait analysis more accurate, efficient and accessible. In this study, we analyzed basic information about gait analysis and AI articles that met inclusion criteria in the WoS Core Collection database from 1992–2022, and the VosViewer software was used for web visualization and keyword analysis. Through bibliometric and visual analysis, this article systematically introduces the research status of gait analysis and AI. We introduce the application of artificial intelligence in clinical gait analysis, which affects the identification and management of gait abnormalities found in various diseases. Machine learning (ML) and artificial neural networks (ANNs) are the most often utilized AI methods in gait analysis. By comparing the predictive capability of different AI algorithms in published studies, we evaluate their potential for gait analysis in different situations. Furthermore, the current challenges and future directions of gait analysis and AI research are discussed, which will also provide valuable reference information for investors in this field.

## 1 Introduction

The term gait describes the characteristics of body movements during walking or running, and the study of bipedal gait in humans, called gait analysis, refers to the objective and systematic study of human movement, including visual observation and instrumental measurement (Theologis, 2011). Gait analysis is a systematic approach that identifies any changes in gait patterns and tries to find out what causes them and how do they affect humans (Perry and Burnfield, 2010). Gait is a complex process achieved through the coordinated movement of body parts, using interactions between internal and external factors, and through the action of the neuromusculoskeletal system (Mirelman et al., 2018). A complete gait process begins when the nervous system gives the command and the muscles pull the bones around the joints. This process requires the division of labor and cooperation of all human systems. Gait involves not only hip, knee, and ankle flexion and extension, but also internal and external rotation, the human center of gravity shift, pelvic tilt rotation and other related movements. It should be noted that if there is a problem in of these inter-related factors, it may lead to an individual abnormal gait. Human gait has certain specificity for various functional abnormalities. Human gait abnormalities lead to specific functional abnormalities. In addition, one of the most reliable indicators of falls is aberrant gait, and poor gait can have additional deleterious effects on mobility and life satisfaction (Melin et al., 2003; Verghese et al., 2010). Therefore, gait analysis is an important tool in clinical practice as it can help to identify specific pathologies and assess disease progression or treatment effectiveness (Celik et al., 2021). Analysis of a person’s gait is essential to determining health status since any apparent variation from normal may point to an underlying disorder.

Traditionally, subjective assessment of gait has been carried out by experienced health professionals. However, with advances in technology, especially the rapid development of artificial intelligence (AI), including the advent of objective and empirical gait analysis, assessments have improved and become more trustworthy. According to its definition, artificial intelligence is a branch of science and engineering that deals with the computational analysis of what is often referred to as intelligent behavior and the development of artifacts that display such behavior. Proficiency in artificial intelligence technology has been explored in almost every field. The challenge for modern medicine is to compile, assess, and employ the enormous body of knowledge needed to deal with complex clinical problems (Ramesh et al., 2004). AI is a subfield of computer science that can examine intricate medical data. In many therapeutic situations, their ability to identify significant associations in data sets can be utilized to make diagnoses, administer treatments, and forecast results. Machine learning (ML) is a field of AI that use statistical algorithms to allow computer systems to progressively improve performance associated with a given job based on data, rather than depending on rules-based programming of the underlying causal linkages (Vikara et al., 2020). Deep learning (DL) is a very new and influential frontier that is a subfield of machine learning (ML) and is based on deep neural networks (DNNs)—neural networks with more than one hidden layer (Chan et al., 2020). Convolutional neural network (CNN), a subclass of DNN, is particularly useful for information identification and classification and has attracted much interest from industry, academia, and clinicians (Chan et al., 2020). Artificial neural networks (ANNs) are a powerful nonlinear modeling technique, particularly effective in gait analysis (Kaczmarczyk et al., 2009). In contrast to other conventional methods, ANN approaches offer the distinct benefit of being non-parametric and requiring little to no prior knowledge of the input data. Due to these factors, ANN approaches may be used in a variety of domains, including pattern recognition, intelligent control, combinatorial optimization, forecasting, and others (Yang and Guo, 2016). Currently, in terms of technology, as the most widely used technique for examining changes in the human movement process, gait analysis in combination with AI has promoted the development of biometric detection equipment and recognition algorithms; In terms of application, the combination of gait analysis and AI can provide guidance for clinical diagnosis, efficacy evaluation, and rehabilitation training, provide solid basic support for the development of biped robots, walking aids, rehabilitation aids and artificial joints. The combined application of gait analysis and AI has become a challenging research topic and will be more extensive in the future.

The further advancement of each particular research direction often requires a review of past research (Berlinberg et al., 2019). Bibliometrics is a kind of useful quantitative science that can track overall research trends in a particular field, and its application in medical research has attracted growing attention (Saab et al., 2019; Zhu et al., 2021). Citation analysis is one of the main tools of bibliometrics. It is of great value to analyze the most cited classical literature to discover the key issues in the research (Moed, 2009). There are few bibliometric studies on gait technology. To the best of our knowledge, no bibliometric analysis of gait analysis and AI has been published so far. Therefore, in this study, we obtained relevant data from articles matched for gait analysis and AI from 1992 to 2022, described the characteristics of the articles, and provided references for a better understanding of the research worldwide. In addition, our research can reveal the limitations and knowledge gaps in the literature to speculate possible research directions. The research will also benefit developers of sensor technology and those interested in remote patient monitoring, which will contribute to the potential improvements of gait-related technology.

## 2 Methods

### 2.1 Study design

To provide scientific analysis, we searched relevant articles from the WoS Core Collection database to identify the articles related to “gait analysis and AI” through systematic literature reviews (SLR). SLR is regarded as a crucial part of the systematic review process, which entails a systematic search of studies with the goal of obtaining a transparent study identification report that provides readers with a clear picture of how the study was identified and how the review’s findings fit into the pertinent evidence (Cooper et al., 2018).

### 2.2 Search strategy

**FIGURE 1:**
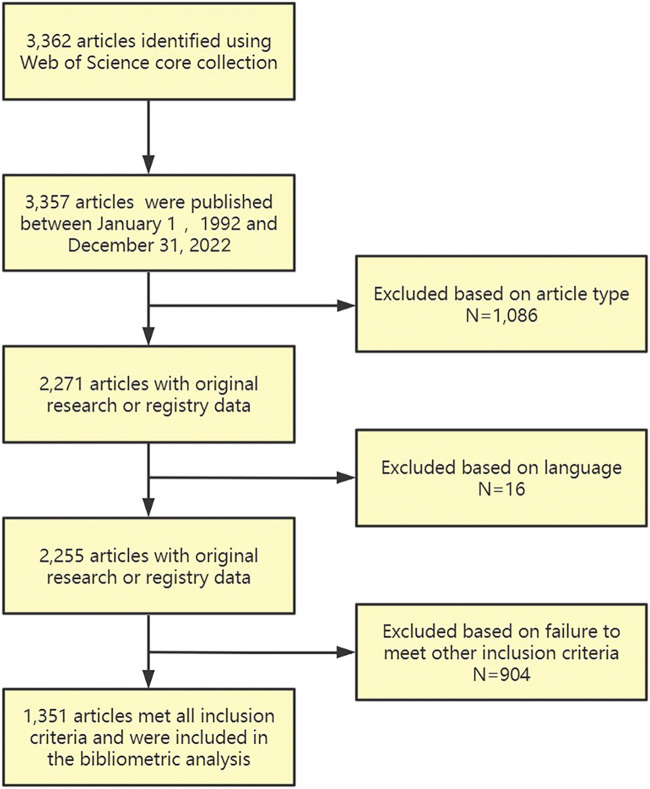
Flowchart of the methodology for identifying articles related to artificial intelligence and gait.

In order to improve the search sensitivity, two researchers (T.B. and J.S.G.) independently selected articles for inclusion. As demonstrated in Figure 1, we identified relevant publications in the WoS Core Collection databases using the specialized search tool, which confined the language and document type to “English” and “Articles or Review Articles” respectively. The ‘front-page’ filter was used to cover only the documents in which search keywords are included in the title, abstract, author keywords, and keyword plus to eliminate the inherent bias of using WoS Core Collection for bibliometric analysis and prevent introducing unrelated publications (Cooper et al., 2018). Furthermore, the field tag, which contains titles, abstract, and keywords, was given as TS= [(“AI” OR “artificial intelligence” OR “deep learning” OR “machine learning” OR “pattern recognition” OR “neural network”) AND (“gait”)], as shown in Table1. According to the inclusion criteria, two researchers (T.B. and J.S.G.) independently examined the abstracts or full texts to find the articles on AI and gait analysis. When the two reviewers disagreed, a third investigator (Y.J.W) joined and helped reach a consensus until the articles were included in the final analysis.

**TABLE 1:** Retrieval function for Gait analysis and AI research.

Retrieval type	Content
Formula	TS= [(“AI” OR “artificial intelligence” OR “deep learning” OR “machine learning” OR “pattern recognition” OR “neural network”) AND (“gait”)]
Language	English
Document type	Articles or Review Articles
Index	All
Date	1 January 1992 to 31 December 2022

### 2.3 Bibliometric analysis

Collect basic information such as journal name, publication date, total citations, average citations per year (ACY), journal name, first author, institution, theme, and keywords. And descriptive statistics of counts or percentages are used to compare different categories of information. The journal impact factors were obtained from the “Journal Citation Reports (JCR)^©^ (2022)” (Clarivate, 2023). There is only the final corresponding author, institution, and country/region in works with numerous corresponding authors. Data mining, mapping and visualization of network analysis was performed using Microsoft Excel and VosViewer (Waltman et al., 2010).

## 3 Results

Bibliometric analysis is a useful tool for retrieving published information and is widely used to quantitatively evaluate academic activity (Sarkodie and Strezov, 2019). Bibliometric analysis can not only be used to explore the characteristics, structure, and development of academic literature, but also can quickly grasp the basic information and research trends in a field. Classical citation recognition is still one of the important methods for the systematic evaluation of scientific research performance. The total number of citations retrieved from the WoS using the SLR was 3,357. When the article type was limited to articles or review articles and the language was English, we obtained 2,255 publications. Finally, 1,351 articles met all inclusion criteria and included in the bibliometric analysis.

### 3.1 Temporal and spatial analysis

**FIGURE 2:**
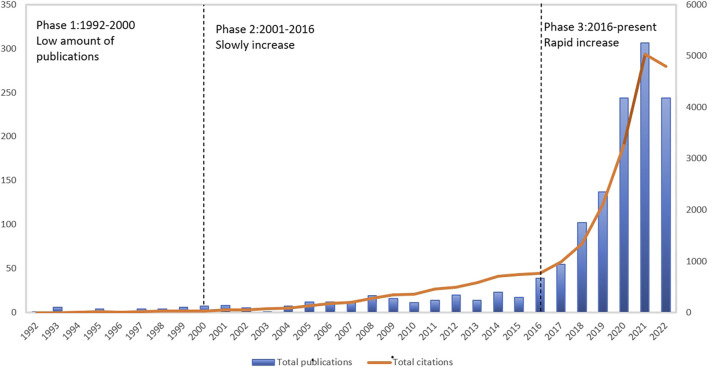
Total publications and citations on AI and Gait during1992–2022.

Figure 2 presents the publishing time trends in terms of both publications and citations. The results indicate a general upward trend in gait analysis and AI research from 1992 to 2022. The three stages below can roughly be used to categorize this trend: 1992–2003: the number of papers published was limited, with gradual increase number of papers published, with a total of 47 papers. 2004–2015: during this stage, the number of publications and citations increased from 47 to 177 and from 2,446 to 8,044 respectively; 2016–2022: in 2016, one study was published in the journal Nature demonstrating the potential of using deep learning algorithms for gait analysis. The study used large-scale gait datasets and utilized convolutional neural networks (CNNs) to identify and categorize various gait patterns (LeCun et al., 2015). The average annual publishing increased to 39–244 documents during this stage of rapid development.

**FIGURE 3:**
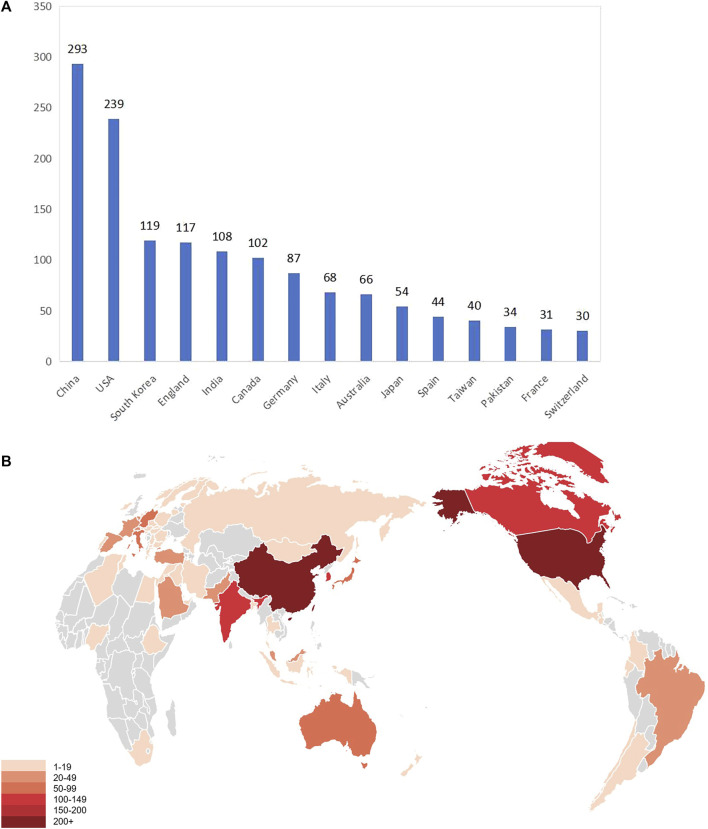
**(A)** The top 15 countries with the highest output of articles **(B)** Geographical distributions of publications during 1992–2022.

There are 76 countries and regions that have published articles, and nearly 11.2% of them have published fewer than 10 articles. Figure 3A shows the top 15 countries with the highest output of articles, and Figure 3B shows the geographical distribution of literature from 1992 to 2022. The results showed that China and United States were in the leading position in the field of gait analysis and AI research. Among them, China ranked first in the number of publications (293 articles, 21.7%), and the H-index was 30; United States ranked second in the number of publications (239 articles, 17.7%), and the H-index was 39. China and United States far outstripped other countries throughout the study period. The performance of Chinese researchers in the fields of gait analysis and AI is noteworthy, especially after the Chinese government issued the *“New Generation of Artificial Intelligence Development Plan”* in 2017, the number of papers published in the field of gait analysis and AI in China increased rapidly. In addition, there were four countries with more than 100 papers: South Korea ranked third, with 119 papers published, and the H-index was 21; The UK ranked fourth, with 117 publications and the H-index was 21; India ranked fifth, with 108 publications and the H-index was 20; Canada ranked sixth, with 102 publications and the H-index was 28. The above eight countries accounted for 78.8% of the papers published in the research field of gait analysis and AI.

### 3.2 The most influential journals

Between 1992 and 2022, 416 journals published the 1,351 papers that were chosen for publication. Only one gait analysis and AI research publication was published in nearly two-thirds of these journals. The top 15 reputable journals for gait analysis and AI research are listed in Table 2, and they accounted for 42.12% of all articles. *Sensors* was the most productive journal, with 162 publication records, followed by *IEEE Access* (72) and *Gait and Posture* (40). *IEEE Journal of Biomedical and Health Informatics* had the highest IF value (7.7) among these top 15 journals. Furthermore, the *Sensors* had the highest TC score (2368), followed by *Gait & Posture* (1562) and *Journal of Biomechanics* (1488). Additionally, in order of the number of citations per publication, *Journal of Biomechanics*, *Gait & Posture* and *IEEE Transactions on Biomedical Engineering* were the top three. According to the results, the core journals were those devoted to gait analysis and AI research, such as *IEEE Access, Gait & Posture, Journal of Biomechanics* and *IEEE Transactions on Biomedical Engineering*. These journals have been the most significant in this subject and are showing a lot of interest in the gait analysis and AI research.

**TABLE 2:** The most productive journals in gait analysis and AI research.

No.	Journal	Country	IF (2022)	TP	TC	TC/TP
1	Sensors	Switzerland	3.9	162	2,368	14.62
2	IEEE Access	United States	3.9	72	791	10.99
3	Gait and Posture	Ireland	2.4	40	1562	39.05
4	IEEE Sensors Journal	United States	4.3	35	462	13.20
5	IEEE Transactions on Neural Systems and Rehabilitation Engineering	United States	4.9	35	565	16.14
6	Journal of Biomechanics	United Kingdom	2.4	29	1488	51.31
7	IEEE Journal of Biomedical and Health Informatics	United States	7.7	26	830	31.92
8	Biomedical Signal Processing and Control	United Kingdom	5.1	23	347	15.09
9	Neurocomputing	Netherlands	6	21	419	19.95
10	Multimedia Tools and Applications	Netherlands	3.6	20	288	14.40
11	Journal Of Neuroengineering and Rehabilitation	United Kingdom	5.1	19	224	11.79
12	Applied Sciences Basel	Switzerland	2.7	18	141	7.83
13	IEEE Transactions on Biomedical Engineering	United State	4.6	18	656	36.44
14	Plos One	United States	3.7	18	629	34.94
15	Scientific Reports	Germany	4.6	17	404	23.76

Note: IF is impact factor of the journal; TP is the number of total publications; TC is the number of total citations; TC/TP is the citations per publication.

### 3.3 Most productive institutions and their collaborations

**FIGURE 4:**
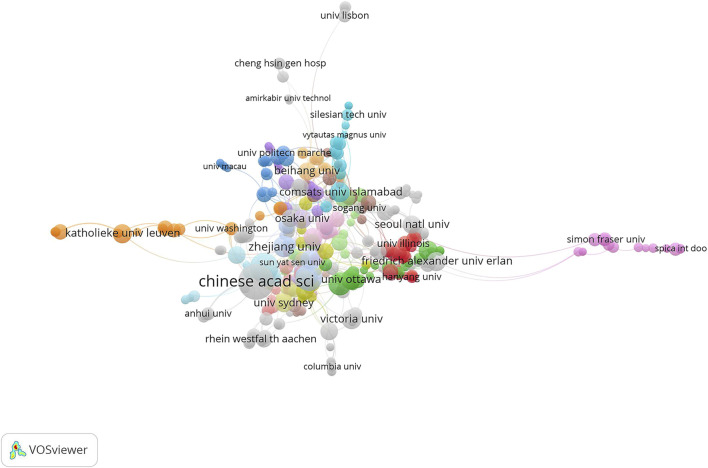
The collaboration network of the most productive institutions.

In this study, a total of 1726 institutions and 1,351 articles were included. Table 3 lists the most influential institutions with more than 13 publications. Of the 13 institutions listed in Table 3, there were three each in China and United States, two each in Canada and Australia, and one each in India, Egypt and Germany. In contrast to the results shown in Figure 3A, UK, South Korea, Japan, and Italy all produced more than 50 papers and were among the most influential countries, but did not have any of the most productive institutions. The Chinese Academy of Sciences has published the most articles, with 41 articles, h-index of 12, and the most cited times of 1,001. In second place was the University of Calgary from Canada, which published 24 articles with 463 citations and the highest H-index (13). The National Institution of Technology in the United States produced 19 articles and was cited 192 times, ranking fourth. It is worth noting that Zhejiang University, ranked 8th, produced 14 articles with 134 citations, also from China. In the top ten institutions of paper output, China has a leading position. The general statistics of the top institutions showed an upward trend, indicating that gait analysis and AI research has developed rapidly in the past years. In addition, Figure 4 shows the close cooperation among these institutions. Institutions in various countries have a close cooperative relationship in the research of gait analysis and AI.

**TABLE 3:** The most productive institutions in gait analysis and AI research.

No.	Institute (country)	TP	TC	RC (%)	h-index
1	Chinese Academy of Sciences (China)	59	1221	20.137	16
2	University of Calgary (Canada)	24	549	23.529	14
3	Xian Jiaotong University (China)	22	307	7.509	10
4	National Institute of Technology System (United States)	19	291	7.950	8
5	University of Erlangen Nuremberg (Germany)	19	674	21.839	13
6	Egyptian Knowledge Bank (Egypt)	17	121	94.444	5
7	University of Waterloo (Canada)	16	426	15.686	12
8	Zhejiang University (China)	14	172	4.778	7
9	Indian Institute of Technology System (India)	13	244	12.037	8
10	Monash University (Australian)	13	645	19.700	9
11	Pennsylvania Commonwealth System of Higher Education (United States)	13	171	5.439	7
12	University of California System (United States)	13	364	5.439	8
13	Victoria University (Australian)	13	821	19.700	9

Note: TP is the number of total publications; RW (%) is the ratio of the total publications of one institute to world publications; RC (%) is the ratio of the total publications of one institute to those of the corresponding country; TC is the number of total citations.

### 3.4 Most contributing authors and their collaborations

**FIGURE 5:**
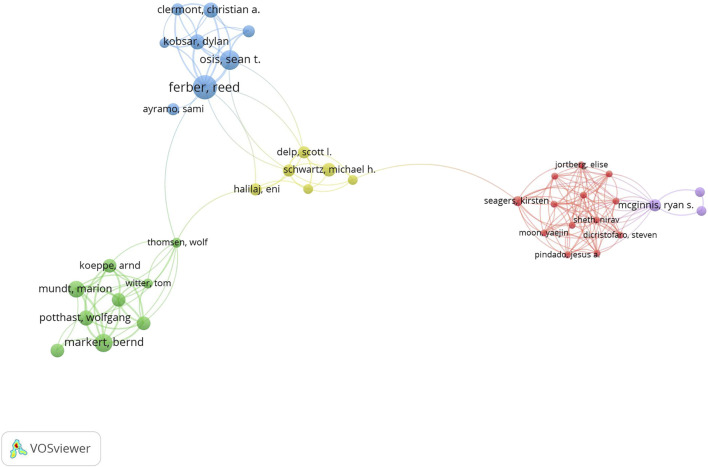
The collaboration networks of the most productive authors.

A total of 4,837 authors participated in research on gait analysis and AI during the past 30 years, and 81% of the authors published only one article. Table 4 lists the top 10 highly productive authors who have published at least 8 papers; This proportion accounted for only 0.3% of all authors, but their publications accounted for 10.36% of the total number of publications. Klucken J from the University of Erlangen Nuremberg published the most articles, with a total of 14 articles and 580 citations; Semwal VB published the most articles as the first author (*n* = 5). Hausdorff JM from Tel Aviv University published 8 articles with the most citations 785 times. The networks of author partnerships on gait analysis and AI studies were analyzed using Vosviewer software in order to investigate the cooperation and collaboration between high-yield authors and other authors (Figure 5). These top-ranked authors form essentially independent research teams and have active collaborative relationships with each other.

**TABLE 4:** The most productive authors in gait analysis and AI research.

No.	Authors	Institution	Position on author list	TC	TP
1	Klucken J	University of Erlangen Nuremberg	First author-1 correspond author-0	14	580
2	Eskofier BM	University of Erlangen Nuremberg	First author-1 correspond author-1	13	415
3	Ferber R	University of Calgary	First author-1 correspond author-4	12	398
4	Lemaire ED	University of Ottawa	First author-0 correspond author-1	12	265
5	Hausdorff JM	Tel Aviv University	First author-1 correspond author-3	8	785
6	Osis ST	University of Calgary	First author-1 correspond author-1	8	335
7	Semwal VB	Indian Institute of Information Technology Allahabad	First author-5 correspond author-4	8	262
8	Kofman J	University of Waterloo	First author-0 correspond author-6	8	248
9	Gassner H	University of Erlangen Nuremberg	First author-1 correspond author-1	8	182
10	Khan MA	NITEC University	First author-1 correspond author-1	8	172

Note: TP is the number of total publications; TC is the number of total citations.

### 3.5 Keyword analysis

**FIGURE 6:**
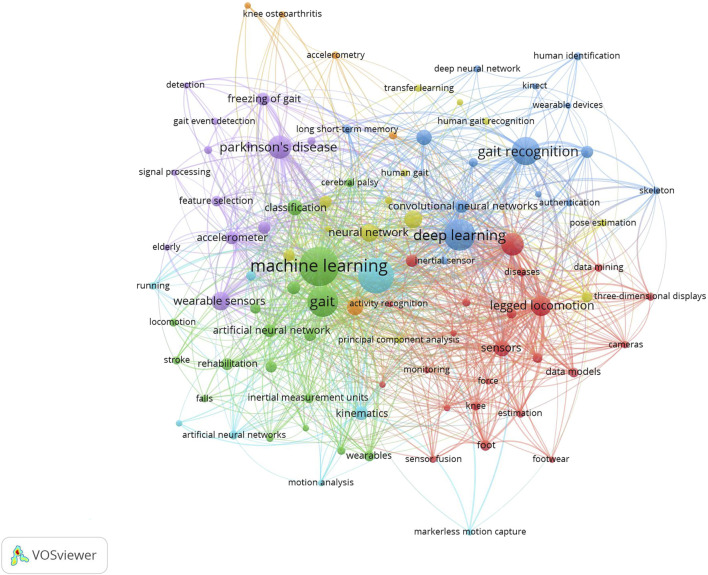
The co-occurrence network of Gait and AI keywords.

Oftentimes, keywords reveal important details about the author’s particular emphasis. Keyword co-occurrence analysis is frequently used to show how terms relate to one another and give readers insight into research hotspots and emerging trends (Mao et al., 2018). In this study, we use keyword co-occurrence analysis to pinpoint the most salient problems and crucial areas for gait analysis and AI research. 3,233 keywords altogether, acquired from 1,351 investigations, are sorted and combined according to gerund, singular or plural type, and abbreviation. The most frequent author keywords were “machine learning”, “gait analysis”, “deep learning”, “gait”, “gait recognition”, “parkinson’s disease”, “feature extraction”, “legged locomotion”, “wearable sensors”, “pattern recognition”, “neural network”, “sensors”, “artificial intelligence” and “convolutional neural network” with over 50 occurrences. The co-occurrence network based on high-frequency terms (more than 7) is shown in Figure 6. The word’s size and center correspond to its magnitude and frequency. Through the analysis of keywords, our study objectively reflects the hotspots of gait analysis and AI research.

### 3.6 Citation analysis

Citation analysis is an important part of bibliometrics and is used to identify and chart the frequency and pattern of citations in literature. Citation times is one of the important indexes to measure the influence of publications. Although this does not always correspond with paper quality, citation counts are thought to indicate the influence of scientific publications (Brandt et al., 2010). Citations may be related to a number of factors, such as the age and accessibility of IF journals (Gao et al., 2020). Articles on gait analysis and AI research were cited 24,189 times from 1992 to 2022, and Table 5 presents the top 10 cited articles. The most cited article was *Decomposing biological motion: A framework for analysis and synthesis of human gait patterns*, which was cited 615 times. Troje, NF was the first author of this article, which was published in *Journal of Vision* in 2002. In this article, researchers create a framework to convert biological motion into a representation that enables analysis using linear methods of statistics and pattern recognition, and they suggest a straightforward motion modeler that can be used to visualize and accentuate variations in walking patterns between men and women (Troje, 2002). The second-most-cited paper presented a multilinear principal component analysis (MPCA) framework for tensor object feature extraction and demonstrated that an MPCA-based gait recognition module achieves highly competitive performance and compares favorably to the most advanced gait recognizers even without a fully optimized design (Lu et al., 2008).

**TABLE 5:** The most cited articles in gait analysis and AI research.

NO.	Title	First author	Journal	Year	TC	ACY
1	Decomposing biological motion: A framework for analysis and synthesis of human gait patterns	Troje, NF.	Journal of Vision	2002	615	27.95
2	MPCA: Multilinear principal component analysis of tensor objects	Lu, HP	IEEE Transactions on Neural Networks	2008	559	34.94
3	Gait dynamics, fractals and falls: Finding meaning in the stride-to-stride fluctuations of human walking	Hausdorff, JM	Human Movement Science	2007	558	32.82
4	A Comprehensive Study on Cross-View Gait Based Human Identification with Deep CNNs	Wu, ZF	IEEE Transactions on Pattern Analysis and Machine Intelligence	2017	317	45.29
5	Self-Powered and Self-Functional Cotton Sock Using Piezoelectric and Triboelectric Hybrid Mechanism for Healthcare and Sports Monitoring	Zhu, ML	Acs Nano	2019	289	57.8
6	Quantification of human motion: gait analysis - benefits and limitations to its application to clinical problems	Simon, SR	Journal Of Biomechanics	2004	289	14.45
7	Support vector machines for automated gait classification	Begg, RK	IEEE Transactions on Biomedical Engineering	2005	237	12.47
8	A review of analytical techniques for gait data. Part 1: fuzzy, statistical and fractal methods	Chau, T	Gait & Posture	2001	236	10.26
9	Sagittal gait patterns in spastic diplegia	Rodda, JM	Journal Of Bone and Joint Surgery-British Volume	2004	219	10.95
10	Automated person recognition by walking and running via model-based approaches	Yam, CY	Pattern Recognition	2004	205	10.25

Note: TC is the number of total citations; ACY is the average citations per Year.

Based on previous studies we can learn that average citations per year (ACY) are a better indicator of an article’s impact and influence on future trends (Tang et al., 2021). We show the top ten articles with the highest ACY in Table 6. *Self-powered and self-functional cotton sock using piezoelectric and triboelectric hybrid mechanism for healthcare and sports monitoring*, published by Zhu, ML as the first author in *Acs Nano* in 2019, had the highest ACY(57.8). In addition, it is the fifth most cited article and the most recently published article among the top 10 most cited articles. Researchers created the S-2-sock in this study to achieve a variety of purposes, including energy harvesting and monitoring different physiological signs (Zhu et al., 2019). Concepts derived from earlier influential articles are assimilated into common sense, reducing citations of the original texts. Studies that were published more recently require more time to build more citations to demonstrate their significance. Articles with high citation counts but low ACY are probably the product of historical accumulation (Tang et al., 2021).

**TABLE 6:** Top 10 articles with the highest ACY.

NO.	Title	First author	Journal	Year	TC	ACY
1	Self-powered and self-functional cotton sock using piezoelectric and triboelectric hybrid mechanism for healthcare and sports monitoring	Zhu, ML	Acs Nano	2019	289	57.8
2	A comprehensive study on cross-view gait based human identification with deep CNNs	Wu, ZF	IEEE Transactions on Pattern Analysis and Machine Intelligence	2017	317	45.29
3	MPCA: multilinear principal component analysis of tensor objects	Lu, HP	IEEE Transactions on Neural Networks	2008	559	34.94
4	Deep learning-enabled triboelectric smart socks for IoT-based gait analysis and VR applications	Zhang, ZX	NPJ Flexible Electronics	2020	138	34.5
5	Gait dynamics, fractals and falls: finding meaning in the stride-to-stride fluctuations of human walking	Hausdorff, JM	Human Movement Science	2007	558	32.82
6	A model-based gait recognition method with body pose and human prior knowledge	Liao, RJ	Pattern Recognition	2020	131	32.75
7	Using smartphones and machine learning to quantify Parkinson disease severity the mobile Parkinson disease score	Zhan, AD	Jama Neurology	2018	185	30.83
8	A review of the evolution of vision-based motion analysis and the integration of advanced computer vision methods towards developing a markerless system	Sharma, Saloni	Sports Medicine-Open	2018	183	30.5
9	Machine learning in human movement biomechanics: best practices, common pitfalls, and new opportunities	Halilaj, Eni	Journal of Biomechanics	2018	174	29
10	Decomposing biological motion: a framework for analysis and synthesis of human gait patterns	Delp, Scott L	Journal of Vision	2002	615	27.95

Note: TC is the number of total citations; ACY is the average citations per Year.

## 4 Discussion

### 4.1 Principles of gait analysis

#### 4.1.1 Gait cycle

**FIGURE 7:**
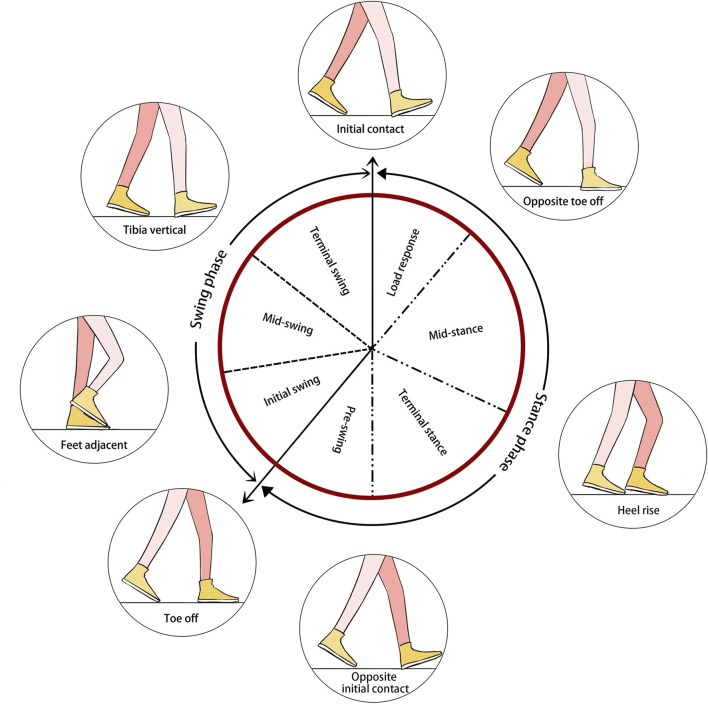
Diagram showing the key phases, stages, and events of the human gait cycle.

The gait cycle represents a series of repeated tasks that culminate in walking. To understand pathology, normal gait patterns are essential to be able to detect alterations in gait. Weber brothers used the concept of the gait cycle and calculated the time of the gait in 1836 (Prakash et al., 2018). The gait cycle is an integrated function of the lower limbs, pelvis, and spine. A gait cycle consists of activity from the initial contact point of one lower limb to the point where the same lower limb contacts the ground again. The limb remains in touch with the ground for around 60% of the gait cycle, which is separated into the support phase’s initial contact, loading response, mid-stance, terminal stance, and pre-swing phases. The swing phase, which makes up the remaining 40%, is broken down into three stages: initial swing, mid-swing, and terminal swing. It is the time when the limb is propelled forward without touching the ground. Gait phase makes it simple to distinguish between the various movement patterns created by individual joints and body segments, which helps with gait analysis (Whittle, 2007). Each gait phase has a distinct objective, and achieving that objective requires a crucial pattern of chosen movement (Sobral et al., 2018). Figure 7 shows the basic gait phases and the expected interval phases and subphases throughout the gait cycle:

a. *Initial contact* The moment the heel of the reference foot made contact with the ground was taken into account. Consequently, it is sometimes referred to as a heel strike. The load response is beginning at this point.
b. *Loading response* It starts when the reference foot makes the first contact and continues until the other foot is lifted and waved in the air. In order to absorb stress, the knee was flexed, and the heel was employed as a rocker. Contact with the forefoot, however, was minimized with the help of ankle flexion, which prevented the heel from acting as a rocker (Tao et al., 2012a). During this time, the supporting limb is fully supported by the torso and the reference foot is fully in contact with the ground.
c. *Mid-stance* It starts with a vertical landing on the swing leg’s tibia. The dorsiflexion of the ankle, which is the rocker arm of the ankle, allows the limb to advance in the stationary foot. The front foot lift and body alignment at the front foot are the two elements of the middle standing phase.
d. *Terminal stance* At this stage, the heel lifts, the forefoot rocker promotes limb advancement, and the body’s weight is transmitted to the front of the forefoot.
e. *Pre-swing* It starts with the other limb’s initial touch and finishes with the toe-off, and its principal role is to position the limbs for swing.
f. *Initial swing* This is the initial swing phase. It starts with lifting the foot off the ground and concludes with the stance foot opposing the swing foot. This phase of flexion limb progression includes increased knee flexion and hip flexion.
g. *Mid-swing* The flexion of the reference foot achieves its maximum extent during this stage, which is referred to as the second phase of the swing.
h. *Terminal swing* The tibia is parallel to the ground during this stage. The tibia swings vertically at the start of this phase, which concludes with the foot’s ball striking the ground. When the lower leg crosses in front of the thigh and the knee extends, limb propulsion is accomplished.

#### 4.1.2 Gait parameters

Inappropriate biomechanics can lead to gait dysfunction, which can lead to serious health problems if not diagnosed promptly and followed up with treatment (Prentice et al., 2001). It would be appropriate to have a prone overview on the parameters that are utilized in gait analysis. Gait data of interest to researchers, particularly physiotherapists and orthopedic surgeons, fall into six broad categories:

a. *Anthropometric parameters* Anthropometric parameters usually take into account the physical dimensions of the human body, including age, sex, weight, height, limb length, and body mass index (BMI). In gait analysis, researchers have argued in favor of grouping together people with comparable anthropometric characteristics. Isolating the effect of anthropometric parameters on gait analysis is essential.
b. *Spatio-temporal parameters* For gait researchers and clinicians, spatio-temporal parameters (TSPs) are often the most understandable and clinically applicable data points (Hecht et al., 2022). The spatiotemporal characteristics of the gait analysis system include step and stride length, step breadth, cadence, velocity, phases (stance and swing), and foot strike and toe-off events. They are among the simplest to assess and correlate with functional severity and disability across a variety of illness conditions, and are occasionally referred to as vital signs of gait (Inam et al., 2010).
c. *Kinematic parameters* By taking into account the motion of the body landmark that was chosen for analysis, joint angles are included. Along with joint angles (such as the angle of the trunk, hips, knees, and ankles), it also includes angular motion, acceleration, and segment trajectory. These characteristics are typically measured using markers and sensors (Sutherland, 2002).
d. *Kinetic parameter* It is a collection of forces that produce ground reaction forces (GRFS). Reverse dynamics enables the estimation of forces and torques for various joints by combining kinematic and GRF data. Total load, weight distribution, including the mapping of the center of pressure and the plantar pressure, as well as joint moments and joint dynamics, are some of these (Hecht et al., 2022).
e. *Electromyography (EMG) parameters* By capturing the timing and force of muscle contractions during the gait cycle, EMG investigations and neuromuscular recruitment analysis can supplement traditional gait analysis. Sequential changes in muscle activation and muscle fiber recruitment can be used to quantify the effects of injury and muscle atrophy on normal gait as well as predict pathological gait abnormalities following surgery (Palmieri-Smith et al., 2013).
f. *Combined parameters* Researchers have made an effort to combine the previously described parameters, such as joint angle and ground reaction force, with anthropometric measures in order to improve analysis and visualization (Lai et al., 2009). By revealing complex correlations between pathological and problematic gait, these studies help clinicians by revealing which patients will benefit most from surgical intervention (Arnold et al., 2006).

The research field determines the factor of interest in the discussed parameters. The choice of precise gait parameters is essential in gait analysis because the choice of the most suitable gait features has a significant impact on the study’s findings.

### 4.2 Gait analysis approaches

Modern methods for analyzing human gait can be broadly divided into four categories: Vision or image processing based using a video camera, sensor-based and other technologies and hybrid approaches (Prakash et al., 2018).

#### 4.2.1 Vision based approach

In methods for vision-based gait analysis, frames are taken with a camera. This analysis can be done in two ways; There are marker-based or markerless on the subject.

Mark-based gait analysis makes use of spherical skin markers with a diameter of 4–25 mm that are either active (light-emitting) or passive (retro-reflective) and are coupled to certain anatomical landmarks or corresponding body segments of the human body via marker clusters or position sensors (Klöpfer-Krämer et al., 2020). The position and orientation of markers in 3D volumes are often determined by optical motion tracking systems, which typically employ near-infrared technology and call for at least two cameras (Baker, 2006; Sander et al., 2012). The camera emits an infrared light signal and detects the reflection of a marker attached to the body. The camera sends out an infrared light signal, which is picked up by the marker’s reflection off the body. Individual markers operate at a specific frequency, therefore this signal is utilized to pinpoint the marker’s location. Current optoelectronic techniques can measure human motion at sampling speeds exceeding 1,000 Hz with spatial resolution up to 1mm, contrary to Winter’s initial belief that a sampling frequency of 50 Hz was sufficient for gait analysis (Winter, 2009b). These contemporary methods offer highly reliable thorough investigation of high-speed motion (Baker, 2006). There are a number of restrictions that must be taken into account, notwithstanding the enormous contribution that marker-based 3D gait analysis has made to patient therapy. Due to the existence of soft tissue aberrations when markers are applied on the skin’s surface, especially in obese patients, the measurement of joint position is frequently incorrect. Although there has been significant advancement, biomechanical models frequently presume that complicated human anatomy is not accurately represented by simplified joints, particularly the knee joint (Szczerbik and Kalinowska, 2011a).

The primary research focus of markerless motion capture, which has its roots in the fields of computer vision and engineering, is the tracking, estimation, and recognition of human motion. Motion capture methods can include background subtraction (Chang et al., 2009), contour extraction (Rigoll et al., 2000; Pratheepan et al., 2009; Muro-de-la-Herran et al., 2014b), shape-from-silhouette methods (Rigoll et al., 2000), optical flow, medial axis transformation or fuzzy clustering process (Corazza et al., 2006; Szczerbik and Kalinowska, 2011b). The data utilized for biomechanical studies or clinical contexts should be accurate and thorough, even if it is possible to provide two-dimensional models with markerless motion capture using just one camera. This necessitates correct modeling of joint mechanics and body motion in 3D models (Mundermann et al., 2006). Thus, multiple synchronized cameras are used in authorized medical equipment built on 3D models. These systems have the significant benefit of allowing measurements to be made in the patient’s environment without the use of specialized laboratory settings. Wearing clothing, carrying a bag or backpack, and having more than one object or other item in the measured space are common contour extraction restrictions. Such questions have been the subject of numerous studies in the past (Knippenberg et al., 2017).

#### 4.2.2 Sensor based approach

Sensor-based approaches offer quantifiable information on physical activity, opening up a variety of applications by detecting parameters such as step number, step speed, step frequency, stride length, foot gap (Patel et al., 2012; Kluge et al., 2018; Brognara et al., 2019), left-right asymmetry, double support, stance and swing time (Brognara et al., 2019), stride variability (Ganea et al., 2012), and activity type, duration, and intensity (Chen and Bassett, 2005). The subject’s body or the ground under them can be equipped with sensors in order to carry out a gait analysis (Tao et al., 2012a; Muro-de-la-Herran et al., 2014a; Ngo et al., 2014). Electromyography (EMG) and inertial measurement equipment were attached to the individual’s body in either a surface or medium based configuration. The dynamics of subject motion were also obtained using the Force Platform.

a. Electromyography (EMG) is a technique for studying muscle electrical activity while walking and detecting gait phase. Motor unit action potentials (MUAPs) were recorded using needle-like or surface EMG electrodes (Sutherland, 2001). The relative muscle tone can also be determined by interpreting the EMG signal’s amplitude during gait, although this needs specialized knowledge of electrode settings and is susceptible to interference (Prakash et al., 2018).
b. Inertial sensors can measure single or multi-point motion trajectories of a subject’s single or multiple body segments during walking. These sensors have become widely used and indispensable for all activities that indirectly or directly address motion because of their lightweight, small size, low power consumption, portability, and low cost (Sprager and Juric, 2015).
c. Floor platform-based sensors are utilized in order to gather the forces that are responsible for the generation of ground reaction forces, force patterns, plantar pressure distribution, as well as step and gait phase recognition. In each step, the foot applies a load to the ground, which pushes back, transmitting a ground reaction force (GRF) to each foot. The magnitude and orientation of the GRF varied during the support phase of each foot and were directly related to the acceleration of the center of mass of the body (Winter, 2009a). The GRF is usually described as vertical forces as well as antero-posterior and mid-lateral shear forces. At each corner of the GRF plate are sensors made of steel plates that measure the force being applied to the plate. The three orthogonal force components of the object’s motion are calculated using this force, which is turned into an electrical signal.
d. Pressure sensors are inserted inside the insole to collect information about the load imposed on the sensor (Alaqtash et al., 2011b). When these piezo-based sensors are subjected to mechanical strain, they generate electrical signals. Researchers can use these methods to identify gait phases, but these techniques have spatial limitations. To make correct measurements, subjects consciously placed their feet in the center of the floor platform so that they could not demonstrate their normal pattern.

#### 4.2.3 Other technologies and hybrid approach

Other technology-based methods for human gait study include electrogoniometers, magnetic resonance imaging, and medical imaging systems. Joint Angle change analysis and step detection can be performed by evaluating the resistance changes of the potentiometers of the two arms in the Electrogoniometer. This method based on Electrogoniometer is not appropriate in time-limited environments due to the fact it takes time to attach to a subject. The magnetic system-based labeling method does not require the line of sight of the marker as the vision-based labeling method does because it uses a magnetic field to track the ferromagnetic marker. It is possible to determine the movements of the surgical segment and the anatomical information of the subject’s surgical segment using methods like magnetic resonance imaging (MRI), computed tomography (CT), and ultrasound. It is then utilized to alter a computational model that may be employed with kinematic and kinetic data (Schöllhorn et al., 2008). But these systems also have limitations that are sensitive to disturbances.

Researchers have improved their understanding of human gait through the use of vision, electromyography, and force platforms (Prentice et al., 2001; Heinen and Osorio, 2006; Schöllhorn et al., 2008; Zhang et al., 2014). Deep learning models offer new options for the detection, fusion, and classification of varied multi-source, multi-sensor data since they require minimal pre-processing on complex data and can produce quicker and more accurate results from a rising variety of databases (Alharthi et al., 2019). Therefore, in addition to the ways mentioned above, there is a hybrid method that combines two or more of the methods mentioned above.

### 4.3 Evolving technology for analyzing gait

#### 4.3.1 Wearable techniques

The miniaturization of sensors, the extension of the field of use for motion tracking systems, and, most crucially, the rise in consumer demand for wearable activity trackers are all results of ongoing technical advancements. This has led to a steady increase in the supply of sensor components as well as a reduction in cost. Therefore, as a consumer product, the market for wearable technology has enormous potential. 115 million units were sold globally in 2017, but by 2023, the market is projected to reach 279 million units (Klöpfer-Krämer et al., 2020). The fields of ergonomics, sports, and medicine can all benefit from the new opportunities afforded by wearable technologies to assess motor function and performance. The continued development of technology has led to the miniaturization and lightening of wearable sensors, which has made possible the expansion of gait parameter measurement outside the confines of laboratory settings (Muro-de-la-Herran et al., 2014a; Shull et al., 2014; Washabaugh et al., 2017).

Wearable sensors mainly include inertial measurement units (IMUs), insole pressure sensors (IPS), electromyography (EMG) sensors, angiometers, inclinometers, electromagnetic trackers, and stretch sensors. However, we were only able to identify three main types of wearable sensors by quantitative analysis of the literature in this study: IMUs, IPS, and a combination of the two.

##### 4.3.1.1 Inertial measurement units

By using threshold or statistical classification systems, acceleration-based techniques, also known as inertial measurement units (IMU), can recognize postures and categorize everyday motions associated with a person’s functional state. Threshing-based motion classification distinguishes between active states using a hierarchical algorithm framework (Yang and Hsu, 2010). Among wearable sensors, Magnetic and Inertial Measurement Units (MIMUs) are the most promising (Yang and Hsu, 2010; Bergamini et al., 2014). They provide several combinations of inertial sensors such as acceleration sensors, gyroscope sensors, and magnetometers (Sang et al., 2018). Evaluation of joint and segmental kinematics is possible with the simultaneous use of complicated and many wearable devices. Therefore, it is essential to understand if the joint kinematics analysis uses an absolute or relative 3D orientation of the inertial sensor to the inertial reference (Chen and Bassett, 2005; Camomilla et al., 2018). All sensors must be sampled at a frequency that is at least twice as fast as the fastest measurable motion in order to assure uninterrupted sampling (Chen and Bassett, 2005).

Researchers have experimented with a variety of methods for gait-related studies by inserting IMUs in various body segments or combinations thereof. There are numerous options for sensor placement. Li et al. (2016) compared the “energy of acceleration” signals from the thigh, calf, and foot, which is the standard for raw acceleration less gravity. They suggested that the “acceleration energy” of the foot seemed relatively more “stable” when graphically examined compared to the other two parts of the body, and therefore recommended that the IMU be placed on the foot. In the context of freezing gait, Mazilu et al. (2012) showed detection performance of 98% or greater for all three body segments, indicating that the question of ideal sensor placement is unimportant. A hidden Markov model (HMM)-based classifier was the subject of a similar observation made by Taborri et al. (2014). They found that employing angular velocities of the foot improved the accuracy of the HMM-based classifier for gait event recognition over using angular velocities of the thigh or calf.

The raw signal from the IMU is noisy, especially the accelerometer signal, so filters are widely used. The two most popular sensor fusion techniques for estimating imu azimuth are those based on Kalman filters and complementing filters. The accuracy, computational cost, and energy efficiency of the two methods were evaluated by Casamassima et al. (2014), who came to the conclusion that the Kalman filter-based approach was the better option. Two of the most well-liked approaches utilizing complimentary filters have been developed by Mahony et al. (2008), Madgwick et al. (2011). Overall, it is well known that the Kalman filter-based approach is more precise but computationally challenging, whereas the complementary filtering approach is known to be computationally light and quite accurate.

Using extra constraints from the interaction of the foot sole with the ground during walking can increase the accuracy of orientation (and position) estimates using the IMU. To account for drift, the zero-velocity update (ZUPT) method or its variations are frequently utilized. The algorithm takes advantage of the fact that the support foot is quasi-static during a certain part of the support phase. At this instant, the linear and angular velocities of the feet are assumed to be zero, and the drift error resulting from the integration is reset. Yang et al. estimated the standing time according to the threshold set by angular velocity and acceleration, which is helpful for the correct application of ZUPT (Lin et al., 2017).

##### 4.3.1.2 Insole pressure sensors

The center of pressure (COP), as well as other gait parameters like step count, gait cycle duration, swing duration, stance duration, and foot-ground interaction events like heel strike (HS) or toe off (TO), are all estimated using insole pressure sensors (IPS), which measure foot pressure distribution (Nguyen and La, 2016). Several IPS variations based on photoelectric sensors, force sensing resistors (FSRs), capacitive sensors, and piezoelectric sensors based on polyvinylidene difluoride (PVDF) film are available (Harle et al., 2012). PVDF films lack endurance despite being trustworthy and affordable. FSRs, on the other hand, are incredibly robust, adaptable, and affordable. FSRs performs well in detecting temporal information, such as the instant of force application, but is less accurate when estimating force magnitude in real time. It can be regarded as the industry standard for wearable sensing because it is the sole wearable sensor used in validation studies (Stoggl and Martiner, 2017).

Due to its low cost, wearability, and unrestricted motion, which permits natural gait in both indoor and outdoor contexts, IPSs is typically viewed in validation studies as an alternative to force plates. Despite these benefits, there are several restrictions that should be taken into account. Since IPSs are typically worn within shoes, they are sensitive to pressure between the shoe and the foot. As a result, pressure readings may not be zero even when the foot is in the swing phase (Senanayake and Senanayake, 2010; Harle et al., 2012). Although IPSs estimate temporal properties similarly to force plates, their use for real-time ground response force estimation is not suggested because it takes significantly longer to achieve the set point than force plates (Harle et al., 2012). Sensor placement, unlike IMU, is not a difficult problem for IPS. While the IMU can be put anywhere on the subject’s body, the IPSs are almost always placed in the subject’s shoe, in the same position as the foot. FSRs are traditionally placed within the IPS at specific hot sites such as the heel, toe, and first and fifth metatarsal bones. This IPSs demands that the subject’s foot proportions be correct so that the FSRs are aligned with the relevant hot spot. Senanayake et al. found measurement errors as a result of respondents with varying foot sizes (6–11), whereas IPS was at a fixed size (8) (Senanayake and Senanayake, 2010). Lin et al. (2016) reported using the derivative of the pressure signal, robustness to this offset, caused by the mismatch between the IPS and the size of the foot. Compared to the traditional approach, which involved placing a number of FSRS on carefully chosen hotspots, the authors’ use of an array of 48 pressure sensors provided better resolution. As IPSs resolution improves, this approach is moving toward placing as many sensors as possible inside the insole to collect data throughout the foot and identify hotspots during signal processing, rather than at the hardware end. When used in real time, this requires more communication bandwidth and computing power to process additional information.

##### 4.3.1.3 Combination of IPS and IMU and other wearable sensors

IPS paired with IMU solutions are emerging in the wearable sensor industry. Examples include Stridalyzer from Retisense in Bangalore, India, Moticon Science from Moticon GmbH in Munich, Germany, and Arion wearable from ATO-GEAR in Eindhoven, the Netherlands (Prasanth et al., 2021). An arrangement like this can combine the benefits of both sensor kinds. Depending on the product, the IMU’s position in relation to the IPS might be fixed, avoiding errors brought on by variations in the IMU’s position among datasets, subjects, and segments.

Electromyography (EMG) sensors, rotary encoders, laser rangefinders, flexion sensors, and capacitive calf orthotics are additional wearable sensors for gait analysis. All other sensors take kinematic measurements aside from the EMG sensor. However, EMG sensors, which monitor muscle electrical activity, have the intrinsic benefit that the signal manifests before the corresponding movement of muscle activation (Duan et al., 2017). Fleischer and Hommel (2008) reported that the EMG signal appeared 20–80 m before the onset of contraction. This would facilitate early perception and thus reduce the latency of control. Farmer et al. (2014) proposed an autocorrelation model that uses EMG signals as input to predict ankle angles, which is said to predict about 100 m in advance. However, there are limitations in the availability of EMG sensors. First, to increase the signal-to-noise ratio, the skin is often shaved, covered with an abrasive gel, and the sensor is attached to the skin to ensure consistent contact and minimize motion artifacts. Second, EMG signals from people with certain impairments, primarily neurological problems, may be weaker and more challenging to understand since EMG signals require more preprocessing/filtering. Additionally, EMG-related characteristics vary from person to person and may frequently change in reaction to alterations in the skin’s and body’s physiological states, such as sweating. The proper placement of the sensor is also crucial and necessitates some training because it should be as near as possible to the relevant abdominal muscles. For less experienced users, this strategy might not be as effective. Most typically, classification algorithms and less frequently, physiological models are used in the evaluation of EMG patterns (Fleischer and Hommel, 2008).

#### 4.3.2 Machine learning techniques

Machine learning (ML) is widely used in many fields such as medical diagnosis (Begg and Kamruzzaman, 2006; Farah et al., 2019), pattern recognition (Shim and Lee, 2015; Souza and Stemmer, 2018), image processing (Leightley et al., 2017; Wei et al., 2018), classification (Van Gestel et al., 2011; Senanayake et al., 2014), predictive analysis (Yoo et al., 2013; Pla et al., 2017; Xiong et al., 2019), monitoring (Van Gestel et al., 2011; Yoo et al., 2013; Senanayake et al., 2014; Xiong et al., 2019; Zeng et al., 2020), and is therefore suitable for gait research. Nonetheless, ML techniques have been used in many gait applications, such as diagnosing gait disorders (Alaqtash et al., 2011a; Devanne et al., 2016; Leightley et al., 2017), predicting early intervention related to fall-related risks due to disability or aging (Begg et al., 2005; Begg and Kamruzzaman, 2006; Paulo et al., 2019), determining motor recovery tasks (Costa et al., 2016b; Goh et al., 2018), or planning rehabilitation or therapeutic interventions (Liu et al., 2016; Thongsook et al., 2019).

The goal of ML in gait analysis is to create a model of a biomechanical system T(x) by establishing the association between input data f(x) and output y(x), despite the fact that the input data is distorted by noise n(t), necessitating pretreatment of the input data (Khera and Kumar, 2020). The initial multidimensional array of input data contains multiple subjects or their trails as ui, and data attributes such as kinematics, kinetics, or neuromuscular signals as vi. The model’s output is a classification of gait abnormalities, events, and activities. The input dataset is split into a training set, test set, and validation set for the iterative process used to evaluate the biomechanical system T(x) using ML approaches. The model was trained using the training set and verified to ascertain the level of fitting once a certain ML technique was chosen. An unreleased test dataset was used to evaluate the performance. The process ends when the correct accuracy is reached; if not, the model parameters must be returned and retrained to get the necessary accuracy. The system becomes difficult when there are too many parameters, therefore feature selection is possible. The most frequently employed techniques are supervised, unsupervised, and reinforcement learning (RL).

##### 4.3.2.1 Supervised learning

The feature vector in this type of learning is made up of labeled data with the intention of finding the optimum function to map the relationship between the input feature vector and the related label. Support vector machines (SVM), neural networks (NNs), random forests (RF), hidden Markov models (HMM), ensemble learning, k nearest neighbors (kNN), and decision trees (DTs) are some of the strategies investigated in gait research. Due to SVM’s high generalization capabilities, even with little datasets, gait analysis has becoming increasingly popular. It can handle both linear and nonlinear problems at its core. Gait research greatly benefits from classification performance that can be expanded to multiple classifications rather than just binary classification (Williams et al., 2014; Guo et al., 2017). The usage of NNs made up of single or multiple layers of perceptrons is the method most frequently utilized in gait analysis. NN employs feedforward and backward propagation methods, which frequently function as a “black box” and do not require manually created features. NN was frequently employed in gait studies to address issues with pattern recognition and prediction. DT is a subtype of RF for extremely nonlinear and complex variable connections. In addition to being interpretable, it is incapable of providing optimal answers. The random forest, on the other hand, chooses the prediction with the most votes out of a group of random DTs. The kNN classifier based on a distance metric is popular in real-time applications since it does not require underlying assumptions about the distribution of the dataset (Costa et al., 2016a; Souza and Stemmer, 2018). In order to account for linguistic information that cannot be stated mathematically, fuzzy techniques are applied in gait asymmetry investigations (Senanayake et al., 2014; Semwal et al., 2015). Due to difficulties specifying several linguistic factors and selecting membership functions optimally, such strategies have not been as thoroughly investigated in gait research.

##### 4.3.2.2 Unsupervised learning

Unsupervised learning eliminates the need for labels because there are no labeled data sets accessible. To provide the required result, the algorithm must independently determine the relationship (Yuwono et al., 2014). Distance plays an important role in clustering. Usually, if the data are close to each other, they cluster into a class. This technique has rarely been explored in gait analysis research, as defining the learning objectives precisely and dealing with a large number of feature vectors becomes a tedious task (Senanayake et al., 2014). However, this technique can be applied when it is unclear how one observation relates to another. To handle large datasets, classifiers need to be combined with some dimensionality reduction methods. These unsupervised techniques are able to learn different patterns of specific diseases. Explanatory studies can ensure that an appropriate distance measure is chosen for a given problem. In addition to distance measures, the classification of subgroups can also be done via latent profile analysis of clusters.

##### 4.3.2.3 Reinforcement learning

Reinforcement learning requires interaction with the system and multiple devices such exoskeletons and walking assistive devices to adapt to a dynamic environment. These devices are employed in the rehabilitation process. In gait rehabilitation, Deep Neural Network (DNN) and RL are frequently employed. For gait rehabilitation, numerous control methods have been created (Hasson et al., 2015). Given its capacity to better capture participant variability and automate in accordance with the requirements of certain objects, RL and deep neural networks (DNNs) are frequently utilized in rehabilitation equipment. Techniques for feature selection and extraction are employed to enhance processing power, while a dimensionality reduction approach is utilized to reduce complexity. Feature selection is the process of selecting the suitable feature and trimming the remainder while maintaining the originality of the feature (Gil et al., 2019). The mathematical procedure of extracting fresh characteristics from existing features is known as extraction. The selection and extraction of features can be automated using classifiers like convolutional neural networks (CNNs), artificial neural networks (ANNs), and deep neural networks (DNNs).

#### 4.3.3 Brain–computer interface techniques

Brain-computer interface (BCI) development has been critical in the study of musculoskeletal gait and brain dysfunction problems in recent years. The premotor and supplementary motor areas (SMA), where motor programs are formed, are activated by sensory inputs from the cerebral and sensory cortices (Khan et al., 2021). The cerebellum is thought to control gait “error/correction” to coordinate appropriate movement by reacting to anomalies in posture (Cunningham et al., 2010; Takakusaki, 2013). Depending on variables like age, weight, and height, BCI technologies behave differently during bipedal movements (Samson et al., 2001; Mahlknecht et al., 2013). A brain-computer interface (BCI) is a communication system that offers users control channels separate from the brain’s output channel so they can use brain activity to control external devices (Nijboer et al., 2008; Nicolas-Alonso and Gomez-Gil, 2012). A standard BCI system has five stages. The acquisition of brain signals using a neuroimaging modality is the first stage. Pre-processing such signals is the second stage since they have physiological sounds and motion artifacts (Pinti et al., 2019). The third stage, known as feature extraction, involves choosing useful traits (Nazeer et al., 2020). Then, appropriate classifiers are used to categorize these traits. The application interface is the last stage, in which the categorized BCI signals are sent as a command to an external device (Moore, 2003).

In various gait applications for BCI, various brain signals such as functional magnetic resonance imaging (fMRI), magnetoencephalography (MEG), electroencephalogram (EEG), or functional near-infrared spectroscopy (fNIRS) are used. In order to analyze the variations in cerebral blood flow and under-neuronal activities for gait analysis, MEG and fMRI provide great spatial and temporal resolution. They are ineffective for gait research in real time, however, because they are not portable (Morshed and Khan, 2014). Non-invasive and transportable brain signal modalities are practical technologies for the analysis of gait abnormalities in online BCI applications. The non-invasiveness, portability, and ease of use of EEG and fNIRS are making them more and more popular in the scientific community. EEG is a neuro-imaging technique with a high temporal resolution that is frequently used for research on gait (Lazarou et al., 2018). In comparison to EEG, fNIRS is a relatively novel technique that was successful in collecting brain hemodynamics. The variations in oxygenated hemoglobin (HbO) and deoxygenated hemoglobin (HbR) during gait can be recorded to aid (Herold et al., 2018). In many applications that cause a hemodynamical response, such as motor rehabilitation, it is essential (Khan et al., 2018). To better understand the brain signals, however, the merging of these several modalities of brain signals can offer supplementary data. As a result, hybrid BCI (hBCI), a new sub-field of BCI, emerged. In hBCI, at least one brain modality is combined with another non-brain data acquisition modality (Pfurtscheller et al., 2010; Hong and Khan, 2017; Hong et al., 2018).

Eliana et al. utilized a treadmill to capture 87% accurate EEG-based walking brain signals for sensorimotor applications (García-Cossio et al., 2015). Lower-limb movement for gait rehabilitation was observed using fNIRS signals by Rea et al. (2014). Perrey investigated neural gait control using fNIRS, focusing on the appropriate cortical regions (Perrey, 2014). EEG-based walking-intention signals were recognized with 82% accuracy in stroke patients by Sburlea et al. (2015). According to their results, patients who were strongly driven to complete rehabilitation-related tasks had a greater success rate. A bipedal robot prosthetic controller was proposed by Zhao et al. (2017). A walking gait pattern was discovered for the robot mechanism, and an online optimized trans-femoral prosthesis control approach (control Lyapunov function (CLF)based quadratic programming (QPs) with variable impedance control) was tested on the prosthetic device’s knee and ankle joints.

#### 4.3.4 Clinical application of gait analysis

Although gait is a complicated process requiring normal musculoskeletal function and being regulated and controlled by the neurological system on several levels, clinical gait assessment is a focused, straightforward, and affordable technique (Jarchi et al., 2018; Mirelman et al., 2018). Gait testing is currently carried out in a minimal amount of time, in a variety of settings, and frequently without the use of expensive devices. Clinicians frequently utilize functional assessments based on behavior or observational gait assessment techniques to gauge their patients’ walking capacity. Researchers are focusing more and more on the study of various gait disorders and gait-related clinical diseases as a result of the rapid development of AI in the field of gait analysis. Low-cost technologies, such as wearables and accelerometers, are being used to objectively quantify gait in clinical practice. According to the characteristics of each disease, gait disorders are usually classified into several types: spastic gait, paralytic gait, ataxic gait, Parkinsonian gait, disturbed gait, involuntary movement, combined gait, and psychogenic gait disorders (Shibasaki, 2010). Clinical decision-making is aided by gait classification, which allows doctors to separate apart gait patterns into groups that have clinical significance (Dobson et al., 2007).

Gait analysis’s therapeutic significance was originally established in the treatment of cerebral palsy in youngsters (Sutherland et al., 1980; Gage, 2004). Motion analysis labs were created as a result of the complicated walking patterns of these kids, the interaction of various joint and muscle issues, and the unpredictable results of orthopedic surgery. As knowledge of gait disorders has grown, various surgical techniques have been created or improved (Ounpuu et al., 1993; Seniorou et al., 2007). Gait analysis is currently an element of preoperative planning for one-stage, multi-level surgery for diplegia and more complicated hemiplegia in the majority of institutions treating children with cerebral palsy. There is still ongoing debate on the repeatability of gait data interpretation; yet, there is evidence that demonstrates how the appropriate application of gait analysis leads to improved outcomes (Graham and Harvey, 2007). After Alzheimer’s disease, Parkinson’s disease is a frequent neurological condition. Assessing the reliability of “gait variability during continuous and intermittent walking in elderly and Parkinson’s disease” was the primary objective of the research conducted by Galna et al. (2013), as well as defining the best count for a sufficient level of certainty regarding gait. Parkinson’s disease has been categorized using “acceleration measurements based on digital gait features” by Rehman et al. (2020). In patients with cerebellar problems, a link between gait variability and falls has been hypothesized (Galna et al., 2013). Research findings have demonstrated a positive correlation between elevated levels of gait variability in the anteroposterior direction regional units and an increased likelihood of falling in individuals diagnosed with cerebellar ataxia. The correlation between spatio-temporal, kinetic, and kinematic gait parameters in COPD patients was investigated by Zago et al. (2018) using the 6MWT, step width, and step length variability as assessment methods. With the aid of gait dynamics, Kaur et al. (2021) looked into the multiple sclerosis prognosis. Gait data extraction took place on a treadmill. Height, weight, age, and gender were employed as normalization variables along with gait metrics. 94.3% was the highest categorization accuracy. Better gait analysis findings can be obtained using machine learning algorithms, which is beneficial for clinical applications like disease tracking or classification. Jun et al. (2020) proposed a classification method based on gated cyclic unit (GRU) classifier and 3D skeletal joint data. The depth information was used to generate a 3D skeleton model. A machine learning model was used to predict the stability data under abnormal gait. In myelodysplasia, gait analysis has also been employed extensively (Duffy et al., 1996). Analysis is typically recommended for the detection of severe valgus knee strain (valgus thrust) and the distinction between coronal and transverse abnormalities, particularly around the hip. Long-term gait monitoring may soon also make use of smartphab-based technology. Ongoing attempts are being made to collect high-quality data from smart, off-the-shelf gadgets that are already commonly used in daily life (Mirelman et al., 2018).

The state of gait analysis techniques has seen some encouraging advancements. Reproducibility will increase with improved precision, quicker acquisition, and strict clinical protocols. We will be able to combine gait analysis with AI methods thanks to better biomechanical models and custom models.

### 4.4 Future directions

Although great progress has been achieved in the study of gait analysis and AI, its applications are still far from being fully optimized. Future uses for clinical gait analysis include recording human gait, extracting gait patterns, rehabilitation, and identifying gait problems (Bin Altaf et al., 2015). Deep learning and machine learning are being used to provide outcomes that are more accurate (Liu and Sarkar, 2006). Additionally encouraging is the development of wearable robots. Robotic rehabilitation has the ability to extend treatment beyond the therapist’s capabilities and can deliver constant, effective care without wearing the therapist out. Better diagnostic potential is demonstrated by ML-based disease prediction techniques. To train machine learning models and make predictions, gait characteristics can be standardized. Gait has undergone substantial research, although its underlying applications have not yet been thoroughly optimized. Future directions for gait analysis and AI research are discussed as follows:

a. Extrinsic, intrinsic, physiological, psychological, and pathological elements all have an impact on how we walk. Despite their best efforts, the researchers were unable to discover a connection between these influencing factors and typical walking.
b. The existing datasets, which have a large diversity in terms of walking environments, are still insufficient to reliably perform a variety of gait analyses. Nevertheless, we cannot generalize about a particular person’s gait pattern. The number of subjects studied was still not large enough to generalize about the standard gait patterns in particular age groups and genders. Moreover, compared to other biometrics like faces and fingerprints, the number of people who can be identified via biometrics is quite small. The dataset could be considered to be freely biased in terms of gender and age because there were not enough samples.
c. Vision-based image states increase gait recognition’s effectiveness. The effectiveness of the recognition algorithm declines as the external covariation increases (Anwary et al., 2019). The difficult area of research will be the creation of these algorithms, which are not dependent on covariation; Researchers are working in these areas. Covariation can be anything, such clothing, bags, and shoes (Nandy et al., 2016). However, 80% accuracy is the highest that can be attained when identifying fabric invariants. Therefore, more effective and accurate methods are needed to address these issues.
d. Data quality suffers as a result of factors like vibration, the location of the sensors in the pocket, and the movement of the garment (Li et al., 2020). Because the location of the sensors affects the quality of the data utilized for analysis, choosing the right location for the sensors is essential for producing high-quality results. In addition, the exact location of the sensor may change depending on the requirements of the application. Therefore, high-quality work is needed in this area of research to detect the best locations to place sensors so that the identification and monitoring of falls can be improved.
e. Sensor fusion is the process of combining data from various sources into a single data set. The accuracy of the data will be increased when compared to single-source data because this will combine data from numerous sources (Ilesan et al., 2022). In gait analysis, sensor fusion can improve spatial and temporal resolution, improve data integrity and coverage, correct sensor errors and drift, and achieve multimodal information fusion (Sabatini, 2011; Tao et al., 2012b; Dehzangi et al., 2017; Qiu et al., 2018). This technique would be very efficient when developing gait-based applications. However, when performing sensor fusion, technical challenges such as data synchronization, data calibration, and algorithm design need to be considered to ensure the validity and reliability of the fusion results.

## 5 Conclusion

The combination of gait analysis and AI provides valuable insights into the understanding and improvement of human walking patterns. AI technologies, including deep learning and machine learning, offer automated and objective approaches to analyze gait data, replacing traditional subjective evaluation methods. These techniques demonstrate significant advantages in feature extraction, event recognition, and gait parameter estimation from gait data. The integration of gait analysis with AI enables us to obtain more accurate and objective gait assessment results, aiding in clinical diagnosis, rehabilitation training, and physical health monitoring.

Deep learning models, such as convolutional neural networks (CNNs) and recurrent neural networks (RNNs), are capable of learning complex spatiotemporal representations from gait data, enabling accurate recognition and analysis of gait events. Through the combination of deep learning and gait data, an extensive range of applications have been developed, including gait recognition, abnormality detection, and motion analysis. These AI algorithms have showcased remarkable results and improved the accuracy and reliability of gait analysis. In addition to AI algorithms, sensor data acquisition also plays a vital role in gait analysis. Some studies demonstrated high accuracy and robustness in gait recognition tasks, emphasizing the potential of sensor data in gait analysis. It is evident that utilizing sensor data greatly contributes to the effectiveness and success of gait analysis with AI.

Despite the myriad advantages of integrating gait analysis with AI, several limitations exist. One primary challenge is the acquisition and processing of high-quality gait data. Consistent and high-resolution data collection is crucial for accurate gait analysis. Additionally, the diversity of gait patterns and inter-individual differences pose challenges for effective gait analysis. To address these issues, researchers must focus on developing advanced sensor technologies, data collection methods, and processing algorithms to improve the reliability and applicability of gait analysis. Another challenge in gait analysis with AI is model interpretability and explainability. Although deep learning and machine learning models yield high accuracy, their black-box nature often limits their interpretability. To enhance the trust and adoption of AI techniques in gait analysis, it is essential to develop transparent and interpretable models that can provide meaningful explanations for the generated results. Additionally, researchers should focus on addressing the limitations of current AI methods in gait analysis. Improving model interpretability and explainability is crucial, as it enables healthcare professionals and end-users to understand and trust the outcomes obtained from AI algorithms. Developing techniques for feature selection and dimensionality reduction in gait analysis will also contribute to enhancing the efficiency and interpretability of the models. Moreover, collaboration between different disciplines, such as biomechanics, computer science, and healthcare, is crucial for advancing gait analysis with AI. By combining expertise and knowledge from multiple domains, researchers can develop innovative solutions that address the challenges faced in gait analysis, ensuring the translation of research findings to practical applications.

The fusion of gait analysis and AI has revolutionized the objectivity and accuracy of gait assessments. This amalgamation has significant implications in various fields, particularly in modern medicine. The potential of AI, specifically deep learning and convolutional neural networks, has heavily contributed to advancements in biometric detection equipment, recognition algorithms, clinical diagnosis, efficacy evaluation, and rehabilitation training. Advancements in sensor technologies, machine learning algorithms, and computational power will likely lead to more sophisticated and accurate gait analysis systems. This synergy has laid a solid foundation for the development of biped robots, walking aids, rehabilitation aids, and artificial joints. Moreover, collaborative efforts between clinicians, researchers, and AI experts are essential to establish standardized protocols, validate AI-driven gait analysis methods, and translate them into clinical practice. Undoubtedly, the combined application of gait analysis and AI represents an exciting research field with promising future prospects.

## Data Availability

The datasets presented in this study can be found in online repositories. The names of the repository/repositories and accession number(s) can be found below: web of science.
